# Forced Cohabitation during Coronavirus Lockdown in Italy: A Study on Coping, Stress and Emotions among Different Family Patterns

**DOI:** 10.3390/jcm9123906

**Published:** 2020-12-01

**Authors:** Emanuela Mari, Angelo Fraschetti, Giulia Lausi, Alessandra Pizzo, Michela Baldi, Elena Paoli, Anna Maria Giannini, Francesco Avallone

**Affiliations:** 1 Department of Psychology, Sapienza University of Rome, 00185 Rome, Italy; angelo.fraschetti@uniroma1.it (A.F.); giulia.lausi@uniroma1.it (G.L.); ale.18.pizzo@gmail.com (A.P.); michela.baldi@uniroma1.it (M.B.); annamaria.giannini@uniroma1.it (A.M.G.); 2 Department of Dynamic and Clinical Psychology, Sapienza University of Rome, 00185 Rome, Italy; elena.paoli@uniroma1.it; 3 Department of Legal and Economic Sciences, Unitelma Sapienza University of Rome, Viale Regina Elena 295, 00161 Rome, Italy; segretariogenerale@fondbg.it

**Keywords:** quality of life, COVID-19, stress disorder, living together, emotional bond, coronavirus disease

## Abstract

Background: At the beginning of 2020, a pandemic caused by a new strain of coronavirus occurred. On March 9th, the Italian population was forced to lockdown to prevent the spread of this new virus. This event forced families and cohabitants to spend their entire days and weeks in the same physical space, interacting with partners and children with a very different degree of intimacy than in the earlier situation. The present study investigated the effects of being forced to live together on different family patterns, on various dimensions such as stress, coping strategies, time perception and quality evaluation of cohabitation. Method: A total sample consisting of 1750 individuals was recruited through a random sample of probability across the Italian country. Due to the lockdown condition, an online questionnaire was set up; several validated scales were chosen, and some open-ended items were included for the thoughts of the participants. Results: The results showed statistically significant differences between the three family patterns examined. Conclusion: During the forced period of living together, a positive effect could be inferred as given by the presence of children and the collaborative coping strategies that have been adopted; the results have been discussed according to the literature on the topic.

## 1. Introduction

When talking about cohabitation, we refer to the process of sharing an experience, and to “living with” in a common and defined time and space. It can be described as a process that allows individuals, organizations and communities to manage meaningful and stable relationships in a physical and symbolic space [1,2]. Living together can be described according to three levels of relationship: social, organizational and emotional. When we talk about the social level, the relationships of cohabitation refer to the context of civic society, the interactions in the community and, in a broader sense, in the global social context. Studies on social coexistence are various and mainly concern the debate and encounter between different ethnic groups, cultures, religions and political orientations [3,4,5], the effects of immigration and social integration processes [6], conflicts related to tolerance, discrimination or crime [7,8], and the construction of a multi-ethnic and multicultural citizenship [9,10]. At the organizational level, the relationships of cohabitation concern those within the workplace; in this case, individuals do not normally choose each other, and the choice of professional environment is also subject to constraints. The organizational context, on the other hand, is a place wherein many individuals spend a large part of their lives, build relationships and set up shared ways of being together, investing energy, emotions and hopes. The studies that have dealt with areas and dimensions related to organizational cohabitation refer, in particular, to cultures and organizational climate [11,12,13], organizational citizenship [14,15], and organizational well-being and health [16,17,18]. Thirdly, on an emotional level, cohabitation relationships concern those within the family of origin, between parents and children or between relatives within an extended family, or relationships as a couple within marriage or outside of marriage, where the members of the couple share a common project and imagine a future together. Studies on emotional cohabitation address the issue of family relationships between parents and children and between siblings [19,20], the reasons for the success or failure of couples’ relationships [21,22], and living together in peer groups [23]. Other studies have investigated emotional cohabitation in particular situations, such as the complexity faced by families in managing new lifestyles and relationships [24], cohabitation in a family with a seriously ill child [25], and changes in cohabitation following catastrophic events [26].

At the beginning of 2020 an unexpected event occurred: a pandemic caused by a new strain of coronavirus—never previously identified in humans—which took the name SARS-CoV-2 (Severe Acute Respiratory Syndrome–Coronavirus–2) according to the indications of the International Committee on Taxonomy of Viruses (ICTV), which deals with the designation and naming of viruses. Italy, for some weeks, has been the second country in terms of number of infections after China. On 24 February, the first decrees of restrictions and social distancing for schools and shops in two northern regions were approved, restrictions that were extended to the whole nation on March 9 by the Ministry of Health.

This pandemic has deeply altered the rhythms and styles of the emotional, as well as working and social, coexistence of millions of individuals following the measures adopted by the Italian Government to deal with the epidemiological emergency (Decree-Law no. 19 of 25 March 2020; Decree-Law no. 33 of 16 May 2020). Strong restrictions were imposed on the free circulation of individuals, and they were forbidden to leave their place of residence except for proven reasons. This has kept some individuals from reaching their homes and their families; it has interrupted, for a large majority of citizens, sociality and organizational coexistence by forcing smart-working. It has forced families and cohabitants to spend their entire days and weeks in the same physical space, interacting with partners and children with a very different degree of intimacy than in the earlier situation.

The issue of the opportunities and difficulties of living together, already well known in the life experience of all individuals, has acquired new attention in terms of investigating its possible effects on the level of affective coexistence that more than two months of confinement have entailed.

The aim of the research is to investigate, through an online questionnaire, the effects of cohabitation caused by COVID-19 lockdown on different types of living together (partner, partner and children, relatives), on various dimensions such as stress, coping strategies, time perception and quality evaluation of cohabitation.

## 2. Method

### 2.1. Procedure

Data were collected from 1 April 2020 to 30 April 2020. Participants completed a secure online survey, optimized for its use on computers, tablets, and mobile devices. The Qualtrics Survey Platform was used to widely distribute the questionnaire throughout Italy. A non-probabilistic and convenience sampling technique was used, in order to successfully attract as many voluntary participants as possible, motivated by interest and curiosity about the research topic.

The questionnaire was distributed through different channels, such as the authors’ official working platforms, by word of mouth and through social networks. After reading the informed consent, each respondent was able to voluntarily decide to join the research and start answering the digital survey. Maximum confidentiality in the handling and analysis of the responses was guaranteed.

### 2.2. Participants

In total, 1750 participants joined the research; 72.7% were female (N = 1273), and the age ranged from 18 to 85 years (M = 36.81; SD = 13.56).

In total, 24.7% of the sample was from the north of Italy, 57.1% from Central Italy and 18.2% were from the south; 47.3% of the sample was single, 44.1% married, 4.7% in a civil union, 1.6% separated, 1.7% divorced and 0.6% widowed (shown in Table 1).

Regarding the educational degree, 10.1% of the sample had a middle school diploma, 6.1% a professional diploma, 28.8% a high school diploma, 16.1% has a bachelor’s degree, 23.8% a master’s degree, and 14.3% had a post-graduate degree, while 0.8% had a different degree.

In total, 25.7% of the sample were students, 12.1% were unemployed, 58.7% were workers and 3.5% were retired.

### 2.3. Materials

For the purpose of the present study, an online questionnaire consisting of different sections was developed. First, a short summary of demographic data (i.e., age, gender, living conditions), then, the questionnaire included the following measures.

#### 2.3.1. Attitudes and Moods about the New Coronavirus

This scale was developed by the authors in order to investigate the attitudes and moods of respondents about the new coronavirus through items such as, for example, “The Coronavirus is a mysterious and highly lethal virus capable of decimating the world’s population” or “The mass media have generated an exaggerated alarm about the real dangers of the Coronavirus”. The 8 items on the scale were evaluated on a 5-point Likert scale, from “Completely disagree” (1) to “Completely agree” (5).

#### 2.3.2. Perceived Stress Scale (PSS)

This scale is widely used to measure stress perception [27]; it aims to understand how participants perceive their lives as unpredictable, uncontrollable and overloaded, and includes questions about their levels of experienced stress. The 10 items of the PSS ask about feelings and thoughts over the past month; respondents are asked how often they feel in a particular way and all responses are on a five-point answer scale from “never” (0) to “very often” (4).

#### 2.3.3. Semantic Differential

The authors developed a semantic differential (a five points scale) to measure attitudes to forced cohabitation during lockdown. It consists of 13 pairs of bipolar nouns, on which respondents had to subjectively place themselves (i.e., “Calm—Excitement” or “Fun—Boredom”).

#### 2.3.4. Cohabitation Scales

The authors elaborated a sequence of scales for the evaluation of the cohabitation construct with respect to the different housing contexts (positive and negative relationship scale). In particular, three different types of scales were developed to measure cohabitation habits in cases where the respondent lived with their partner, their partner and their children or family members/relatives. For two of the cohabitation scales, 10 items were developed, while the scale on cohabitation with children included 12 items; all were evaluated on a 5-point Likert scale from “Never” (1) to “Very often” (5).

#### 2.3.5. Coping Orientation to Problem Experienced—New Italian Version (COPE-NVI)

This scale evaluates coping styles and asks participants to evaluate the frequency with which they perform specific coping strategies in difficult or stressful situations [28]. The instructions ask participants not to refer to a specific episode, but rather to think about how they usually behave in stressful situations.

In this study only 8 out of the original 25 items were selected; the response alternatives of the chosen items were on a five-point scale ranging from “Never” (0) to “Very often” (4).

#### 2.3.6. Stanford Time Perspective Inventory (STPI—Short Form)

This instrument provides a simple way to measure multiple temporal perspectives of individuals, and is built on a theoretical basis that examines the emotional, social, cognitive and motivational processes that are supposed to contribute to, and are in turn influenced by, the functioning of the temporal perspective [29]. Based on the specific purpose of this study, a selection of the original 22-item scale was made, whereby only 9 items were used, and all the answers were on five-point response scales ranging from “Never” (0) to “Very often” (4).

#### 2.3.7. Future Forecasts

Finally, the authors developed a scale in relation to the future forecasts expressed by respondents. Thirteen items were elaborated (for example, “Overall health management will improve”), with a 5-point Likert scale response from “Completely unlikely” (0) to “Very likely” (4).

#### 2.3.8. Social Lockdown Perception

A single item was formulated to investigate thoughts and moods related to the lockdown; participants were asked to answer the question “Try to define in a few words this period of relational and social life restrictions”.

### 2.4. Data Analysis

Participants were divided into three groups and were compared across some different scales, such as stress, coping strategies, time perception and quality evaluation of cohabitation. After performing basic descriptive analyses, a content analysis for Social Perception was performed. Then, differences between groups were examined using the analysis of variance (One-way ANOVA), and statistical significance in post-hoc analysis was determined using Bonferroni correction. Statistical significance was defined as *p* < 0.05. The IBM SPSS software version 25 was used for statistical analysis.

## 3. Results

### 3.1. Demographic Statistics

As far as concerns the current working condition, 6.3% of the participants were entrepreneurs or managers, 18.7% were qualified professionals (e.g., lawyer, psychologist, accountant), 12.2% were managers or officials, 20.9% were executives, 6.0% were manual workers, 0.3% worked in agriculture, 4% were law enforcement officers, 6.4% were doctors, 12.6% were teachers, and 12.5% were employed in a different profession from those listed above.

The sample consisted of three different family configurations: 27.5% of the participants lived with the partner, 35.4% with their partner and children, while 37.1% lived with their parents or family members, and 48.1% of participants had a pet.

Finally, regarding housing conditions, 5.4% lived in a house of less than 50 sqm, 44.3% in a house between 51 and 90 sqm, and finally 50.3% in a house over 91 sqm. A second question investigated concerned the presence of outdoor spaces in the house. In this case, 40.9% had at least one balcony, 44.1% had a private terrace or garden and 10.1% had a communal terrace or garden, and 4.9% had no outdoor spaces.

### 3.2. Social Lockdown Perception

Regarding Social lockdown perception, participants were asked to freely define through a maximum of three words (not sentences) the lockdown period. It was not required to enter words with a specific positive or negative value.

A total of 7209 terms was recorded and then they were grouped through a double-blind procedure into 1045 clusters (terms such as prepositions, articles, and adverbs were removed); in order to minimize the possible experimenter inferences, no changes were made to the different words. Therefore, no adjectives were changed into nouns and vice versa, but only words with the same semantic root and different desinence (e.g., masculine/feminine, singular/plural) were grouped together.

**Figure 1:**
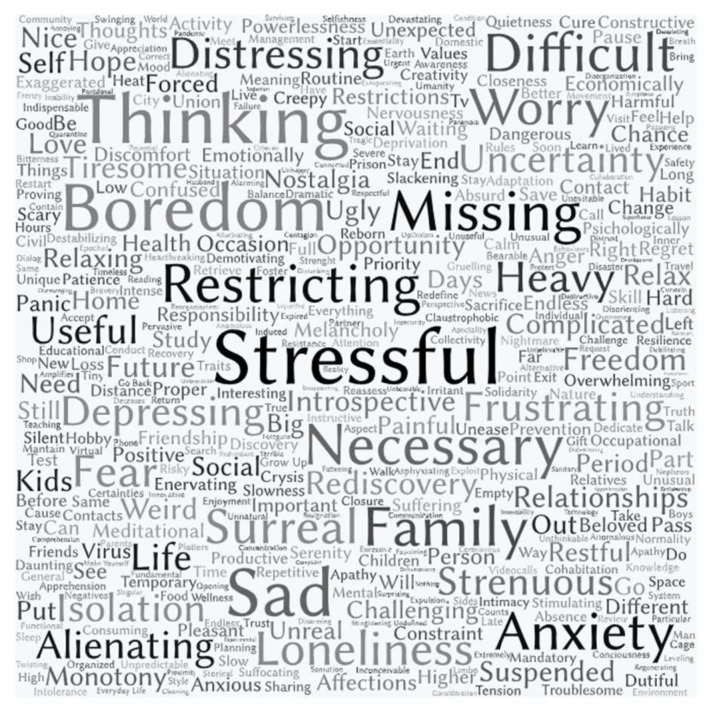
Visual representation of the words related to lockdown social perception.

Frequencies analysis results are shown in Figure 1. The 10 most used words were, in descending order: “stressful”, with 227 instances (31.5%) followed by “thinking”, with 185 instances (25.6%); “sad”, with 177 instances (24.5%); “boredom”, with 172 instances (23. 8%); “necessary” with 131 instances (18.2%); “missing” with 123 instances (17.6%); “family” with 119 instances (16.5%); “restrictive” with 115 instances (15.9%); “difficult” with 111 instances (15.4%) and “anxiety” with 19 instances (15.1%).

It is important to underline that, while the most frequent words have a negative or neutral meaning, the first words with a full positive connotation appeared after position 23, and are: “useful” (0.8%), “rediscovered” (0.7%), “freedom” (0.7%) and “hope” (0.5%).

### 3.3. Semantic Differential

**Figure 2:**
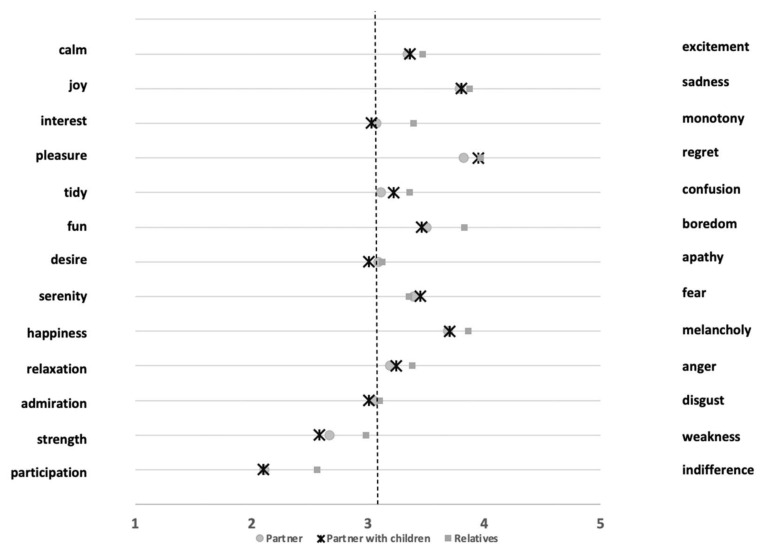
Graphic representation of the semantic differential concerning emotions, group comparison.

Through a semantic differential, attitudes and emotions with regard to forced cohabitation during lockdown were queried. The 13 pairs of bipolar nouns were analyzed using a one-way ANOVA in order to identify any differences between the three groups (Figure 2) through post-hoc comparison. Lower mean values are closer to positive nouns, while higher mean values are oriented towards the negative nouns.

Statistically significant differences have been found, through post-hoc comparisons, between individuals in the three groups. More in detail, in Interest–Monotony, F_(2, 1747)_ = 14.94, *p* < 0.001 between individuals living with relatives (M = 3.39, SD = 1.27) and individuals living with the partner (M = 3.07, SD = 1.26), and between individuals living with relatives and individuals living with partners and children M = 3.03, SD = 1.29. In Pleasure–Regret F_(2, 1747)_ = 3.73, *p* < 0.05 between the relatives group (M = 3.97, SD = 0.92) and partner group (M = 3.82, SD = 1.00). In Tidy–Confusion F_(2, 1747)_ = 6.38, *p* < 0.001 between individuals living with relatives (M = 3.36, SD = 1.18) and individuals living with the partner (M = 3.11, SD = 1.19). In Fun–Boredom F_(2, 1747)_ = 27.12, *p* < 0.001 between the relatives group (M = 3.83, SD = 0.94) and partner group (M = 3.50, SD = 0.96), and between relatives and partners with children M = 3.46, SD = 1.02. In Happiness–Melancholy F_(2, 1747)_ = 6.09, *p* < 0.01 between individuals living with relatives (M = 3.86, SD = 0.92) and individuals living with the partner (M = 3.69, SD = 0.94), and between individuals living with relatives and individuals living with partners and children M = 3.70, SD = 0.98. In Relaxation–Anger F_(2, 1747)_ = 5.18, *p* < 0.01 between relatives the group (M = 3.38, SD = 1.07) and partner group (M = 3.19, SD = 1.08), and between the relatives and partners with children groups M = 3.24, SD = 1.06. In Strength–Weakness F_(2, 1747)_ = 20.82, *p* < 0.001 between individuals living with relatives (M = 2.98, SD = 1.17) and individuals living with the partner (M = 2.67, SD = 1.09), and between individuals living with relatives and individuals living with partners and children M = 2.58, SD = 1.15. In Participation–Indifference F_(2, 1747)_ = 38.50, *p* < 0.001 between the relatives group (M = 2.56, SD = 1.13) and partner group (M = 2.11, SD = 1.01), and between the relatives and partner with children groups M = 2.10, SD = 1.02. In each case, the individuals living with the partner reported positive pole-oriented scores for the adjective couple.

As far as comparisons between individuals living with the partner and individuals living with partners and children are concerned, no statistically significant differences have been identified.

### 3.4. Attitudes and Moods about The New Coronavirus

Regarding the “Attitudes and moods about the new Coronavirus” scale, there are some interesting results. In particular, with respect to the item “The Coronavirus is a mysterious and highly deadly virus capable of decimating the world’s population”, 7.2% declared to “Completely disagree”, 18.3% to “Quite disagree”, 17.4% to “Neither agree nor disagree”, 37.4% declared to “Quite agree” and 19.7% to “Completely agree”. Additionally, the item “The authorities were right in closing bars, restaurants, shops, etc. and inviting the population to stay at home” had some interesting answers, with 70.8% declaring to “Completely agree” and 22.7% to “Quite agree”, while the remaining 6.5% is distributed between “Completely disagree” (1%), “Quite disagree” (2%) and “Neither disagree nor agree” (3.5%). Finally, as regards the item “The Italian population behaved responsibly respecting the limitations imposed by the authorities”, the respondents were grouped as follows: 6% “Completely disagree”, 26.5% “Quite disagree”, 17.7% “Neither disagree nor agree”, 44.3% “Quite agree” and only 5.5% “Completely agree”, showing a general confidence in the population, but also a certain degree of criticism towards some behaviors.

### 3.5. Future Forecasts Scale

Regarding the “Future forecasts” scale, the results show some interesting outcomes. For example, the items related to a “hope” dimension show the general confidence of the population in the possibility that things will improve. The item “Overall health management will improve” reported a 45.5% response of “Probable”. To the item “Overall school management will improve”, 33.9% said that it would be “Probable”, and with respect to the item “Individuals will learn to manage their health better” 38.8% considered it “Probable”. Other interesting items are those related to the “political” dimension (to the item “It will be discovered that the policy of closing cities has been wrong”, 35.1% answered with “Very unlikely” and 39% with “Unlikely”) and to the “corruption” dimension (in particular, the item “Corruption in Italy will decrease” shows that 51.5% believe that this will be “Very unlikely”). Important too are the forecasts made by respondents regarding the items “Separations between spouses will increase” (to which 47.8% responded with “Probable”) and “Domestic violence will increase” (46.2% believe that this will be “Probable”).

### 3.6. Cohabitation Scales: Positive Relationship

A one-way ANOVA was conducted to compare the three groups regarding “Positive Relationship Cohabitation Scales” (shown in Table 2), and statistically significant results emerged on “We had a good level of confidence and complicity”, in which the partner group differed both from partner and children and relatives (F_(2, 1747)_ = 58.49, *p* < 0.001); however, post-hoc comparisons using Bonferroni correction revealed a statistically significant difference between the groups partner (M = 4.11, SD = 0.86), partner and children (M = 3.77, SD = 1.00) and relatives (M = 3.48, SD = 1.03). For the item “In some moments of discomfort, I could rely on her/him/them”, the partner group differed both from partners and children, and relatives (F_(2, 1747)_ = 58.61, *p* < 0.001), and in post-hoc comparisons there was a statistically significant difference between partner (M = 4.16, SD = 1.02), partner and children (M = 3.84, SD = 1.16) and relatives (M = 3.42, SD = 1.27).

There were statistically significant differences between three groups (F_(2, 1747)_ = 64.90, *p* < 0.001) on the item “We have been able to listen to each other and respect our differences of opinion”, wherein the partner group (M = 4.00, SD = 0.92) differed from partner and children (M = 3.74, SD = 0.98) and relatives (M = 3.31, SD = 1.15). Ultimately, statistically significant results emerged for “We have been patient enough to tolerate some annoying character from each other”, in which the partner group differed both from partners and children and relatives (F_(2, 1747)_ = 58.81, *p* < 0.001); however, post-hoc comparisons using Bonferroni correction revealed a statistically significant difference between partner (M = 3.96, SD = 0.83), partner and children (M = 3.75, SD = 0.89) and relatives (M = 3.37, SD = 1.05).

### 3.7. Cohabitation Scales: Negative Relationship

A one-way ANOVA was conducted to compare the three groups regarding “negative relationship cohabitation scales” (shown in Table 3), and statistically significant results emerged on “We have had verbal arguments and disagreements” in which the relatives group differed both from the partners and children and the partner group (F_(2, 1747)_ = 15.34, *p* < 0.001); however, post-hoc comparisons using Bonferroni correction revealed a statistically significant difference between relatives (M = 2.75, SD = 0.89), partners and children (M = 2.61, SD = 0.83) and partner (M = 2.47, SD = 0.83).

Statistically significant results emerged on “Mutual trust has been compromised”, in which the relatives group differed both from partners and children and partner (F_(2, 1747)_ = 16.88, *p* < 0.001); however, post-hoc comparisons using Bonferroni correction revealed a statistically significant difference between relatives (M = 1.83, SD = 1.00), partners and children (M = 1.66, SD = 0.96) and partner (M = 1.50, SD = 0.83).

Lastly, for the item “Our differing views were unbearable”, the relatives group differed both from partners and children and partner (F_(2, 1747)_ = 20.91, *p* < 0.001), and in post-hoc comparisons there was a statistically significant difference between relatives (M = 2.15, SD = 1.11), partners and children (M = 1.93, SD = 1.06) and partner (M = 1.75, SD = 0.96).

### 3.8. Stanford Time Perspective Inventory

A one-way ANOVA was conducted to compare the three groups regarding “Stanford Time Perspective Inventory” (shown in Table 4), and statistically significant results emerged on the item “I believe that my future is beautiful and well planned,” in which the partners and children group differed both from the partner group and the relatives group (F_(2, 1747)_ = 29.98, *p* < 0.001); however, post-hoc comparisons using Bonferroni correction revealed a statistically significant difference between partners and children (M = 2.17, SD = 0.91), partner (M = 1.99, SD = 1.00) and relatives (M = 1.73, SD = 1.10).

### 3.9. Perceived Stress and Coping Orientation to Emotions and Problems

A one-way ANOVA was conducted to compare the three groups regarding PSS (shown in Table 5). The results show statistically significant differences, especially between relatives and the two other groups, partners with children and partners, both with respect to negative items (for example, “In the last month, how often have you felt nervous and «stressed»” (F_(2, 1747)_ = 16.22, *p* < 0.001, with relatives M = 2.41, SD = 0.99; partners with children M = 2.17, SD = 0.90; partners M = 2.12, SD = 0.91)) and positive items (for example, “In the last month, how often have you felt that you were on top of things” (F_(2, 1747)_ = 18.06, *p* < 0.001, with relatives M = 1.82, SD = 0.79; partners with children M = 2.20, SD = 0.82; partners M = 2.13, SD = 0.82)). The results highlight that there is greater perceived stress in relatives in comparison to partners with children and partners, which report lower average scores for all items on the scale. In addition, the three groups were compared with respect to the positive (F_(2, 1747)_ = 47.64, *p* < 0.001) and negative (F_(2, 1747)_ = 16.13, *p* < 0.001) sub-dimensions of the PSS, and post-hoc comparisons using Bonferroni correction showed statistically significant differences in the PSS-negative dimension between partners and children (M = 10.69, SD = 3.95), partners (M = 10.79, SD = 4.24) and relatives (M = 11.9, SD = 4.27). Statistically significant differences were also found in the PSS-positive dimension between partners and children (M = 10.35, SD = 2.28), partners (M = 10.25, SD = 2.41) and relatives (M = 9.11, SD = 2.74). The results therefore show that relatives, compared to partners with children and partners, generally have more negative perceived stress and less positive perceived stress.

A one-way ANOVA was also conducted to compare the three groups regarding “Coping Orientation to Problem Experienced” (shown in Table 6). Once again, relatives showed more general statistically significant differences from partners with children and partners (e.g., “I think about how I might best handle the problem”, F_(2, 1747)_ = 16.93, *p* < 0.001 with relatives M = 2.99, SD = 0.87; partners with children M = 3.24, SD = 0.77; partners M = 3.18, SD = 0.75). Moreover, the three groups were compared with respect to the emotion (F_(2, 1747)_ = 8,45, *p* < 0.001) and problem (F_(2, 1747)_ = 7,79, *p* < 0.001) sub-dimensions of the COPE, and post-hoc comparisons using Bonferroni correction outlined statistically significant differences in the COPE–emotion dimension between partners and children (M = 8.24, SD = 2.46), partners (M = 7.62, SD = 2.49) and relatives (M = 7.65, SD = 2.64). Statistically significant differences were also found in the COPE–problem dimension between partners and children (M = 10.84, SD = 2.23), partners (M = 10.56, SD = 2.29) and relatives (M = 10.32, SD = 2.49). The results show, once again, that relatives, compared to partners with children and partners, report lower average scores when compared to problem-focused coping strategies, while partners with children report higher average scores with respect to emotion-focused coping strategies.

## 4. Discussions

The results of this research allow different considerations on the influence that interpersonal relationships have on the quality of living together, in particular on the emotional level. Several studies report the importance of the type of relationship in living together [19,21]; in this study, we aimed to investigate the possible effect of the relationship as a mediator on stress, future perspectives and coping strategies during the COVID-19 lockdown in Italy.

PSS results showed that perceived stress is greater in the relatives group. In particular, negative perceived stress showed higher scores in the relatives group, instead of the partner and partners with children groups, while positive perceived stress showed higher scores in the partners with children and partner groups compared to the relatives one. Furthermore, an important fact arises from the comparison between the three groups: in general, partners with children showed statistically higher scores in COPE-NVI. In particular, the results showed that the partners with children group engaged coping strategies related to emotions and problem solving more frequently than other groups, suggesting that children could play a moderating role in the implementation of stress coping strategies. Similar results emerged from the STPI; in fact, as far as concerns the future perspective, the group partners with children showed higher values than the other two groups, showing a greater tendency towards planning for future events. These results can be explained through the positive dyadic coping and collaborative coping constructs; these coping constructs consider stress management as a collective and not individualistic process—the subject does not manage stress in a void but within an interpersonal context [30]. Giving support to the partner in stressful situations would increase the quality of the conjugal relationship, but also the physical and psychological well-being of the family members, both in cases of daily stress and in extraordinary stressful events [31,32,33]. In highly stressful situations, significant others, especially the partner, are a great support source, but so too are active participants in family management and the influence of the management process itself [34,35], and activate shared coping strategies [34].

Moreover, according to Berg and colleagues [36], there is a positive association between dyadic coping and partner well-being. More specifically, when partners reported more collaborative coping, they reported more positive emotions. This result seems to be in line with the present study result; in fact, from the data that emerged from the semantic differential on the perceived emotions during lockdown, it could be noted that positive emotions are more likely to be experienced by individuals who live with their partner, and with partners and children.

From the data analysis, an interesting finding seems to emerge from the comparison between the future forecasts scale and the positive and negative relationships scales. According to participants’ future forecasts, as a result of the COVID-19 emergency an increase in spousal separations is likely to occur, as well as in domestic violence. However, according to the positive and negative relationships data, the results seem to be in contrast with the future forecasts scale. In fact, while in future forecasts negative aspects at a relational level (i.e., separations) emerge, on the other side, items from the positive relationships scale, referring to complicity, trust, listening and respect, show above-average scores, both in the general sample and especially in those who reported living with the partner only, and with partners and children. The same thing happens, on the contrary, with the negative relationships scale (e.g., indifference, arguments, differences of opinion, but also verbal and physical violence), on which scores were quite low, in contrast with the risk perception of increasing separations and domestic violence. This apparent contradiction between the data could be explained through fundamental attribution error [37], which is the tendency to underestimate the role of situational determinants and overestimate the degree to which social actions and outcomes reflect the dispositions of relevant actors.

Finally, from the qualitative analysis, a prevalence of negative words and ideas emerges, referring to the experience during lockdown and forced cohabitation caused by the Ministerial Decrees (Decree-Law no. 19 of 25 March 2020; Decree-Law no. 33 of 16 May 2020). Since responses were not sentences and only three words were asked for, it was not necessarily possible to assign a meaning (e.g., positive, negative or neutral) to the terms, but these analysis results allowed a broader interpretation of the emerging data; however, it could be noteworthy that the first word with a fully positive meaning (i.e., “useful”) appeared in the twenty-third position, while the previous words all had a negative (i.e., “stressful”) or neutral (i.e., “thinking”) meaning. From what emerges, most people have reported to experience the lockdown as a difficult period, reporting heavy and meaningful words, often related to strong psychological distress conditions (i.e., alienating, depressing, anxiety, crisis).

Overall, it can be hypothesized that during the COVID-19 lockdown, interpersonal relationships with people living together had an important impact on psychological well-being. An emotional bond seems to be an important mediator in the management of stress and negative emotions; moreover, the COVID-19 outbreak has generated a unique situation with specific characteristics related to stress, coping strategies and living together, creating a new multifaceted form of cohabitation, highlighting the need to create and validate instruments which can be used in unexpected events. New studies could investigate the effects of forms of cohabitation without emotional bonds (e.g., roommates), also by using these ad hoc instruments, in order to validate them for use among the scientific community.

This study may give rise to a consideration for future research considering the possible mediating effect of the caregiver role. In fact, the partners with children group suggested the possible positive effect on stress and coping strategies of being a caregiver; however, those living with relatives could equally be caregivers for both children and elderly or disabled relatives. Therefore, it could be investigated if it is the caregiver role that act as a mediator in the experience of positive emotions—for example, differentiating within the relatives group between those who take care of a cohabitant relative and those who do not—or if it is the presence of children which activates positive emotions.

These results may have important practical and clinical implications—emotional cohabitation plays a key role in stress and coping management, proving to be a good basis for possible counseling interventions, but also for the development of awareness and preventions projects related to COVID-19′s psychological consequences that are also linked to educational programs on living together and emotional relationships. Since lockdown has led to intense stress, fatigue and anxiety [38,39], this kind of study may help identify potential action fields and protocols in order to reduce stress and increase awareness of cohabitant relationships, which will result, in turn, in greater psychological well-being.

## Figures and Tables

**Table 1:** Demographic characteristics of the sample classified by living together patterns.

		Partner	Partners with Children	Relatives
Gender	Male	119	154	204
	Female	362	466	445
Geographic Area	North Italy	167	173	92
	Central Italy	245	321	434
	South Italy	69	126	123
Marital Status	Single	195	52	581
	Married	217	511	44
	Civil Union	48	29	6
	Legal Separation	7	13	8
	Divorced	13	10	6
	Widow	1	5	4
Total		481	620	649

**Table 2:** Positive relationship cohabitation scale: “In the last month, with the partner I live with…”, group comparison. Note: PwC = partners with children; R = relatives; P = partner.

ANOVA										
		Sum of Squares	df	Mean Square	F	Sig.	Multiple Comparison	Mean Difference	Sig.	η^2^
I freely faced every thought or emotion	Between Groups	177.098	2	88.549	94.666	0.000	P vs. R	0.713	0.000	0.10
	Within Groups	1634.116	1747	0.935			PwC vs. R	0.606	0.000	
	Total	1811.214	1749							
We had a good level of confidence and complicity	Between Groups	111.657	2	55.828	58.486	0.000	P vs. PwC	0.342	0.000	0.06
	Within Groups	1667.614	1747	0.955			P vs. R	0.635	0.000	
	Total	1779.271	1749				PwC vs. R	0.293	0.000	
We have divided up the household management tasks equally	Between Groups	92.066	2	46.033	36.949	0.000	P vs. R	0.541	0.000	0.04
	Within Groups	2176.506	1747	1.246			PwC vs. R	0.397	0.000	
	Total	2268.571	1749							
In some moments of discomfort, I could rely on him/her/them	Between Groups	159.002	2	79.501	58.613	0.000	P vs. PwC	0.326	0.000	0.06
	Within Groups	2369.569	1747	1.356			P vs. R	0.748	0.000	
	Total	2528.571	1749				PwC vs. R	0.423	0.000	
Together we faced the difficulties	Between Groups	142.670	2	71.335	62.978	0.000	P vs. PwC	0.188	0.011	0.07
	Within Groups	1978.815	1747	1.133			P vs. R	0.677	0.000	
	Total	2121.486	1749				PwC vs. R	0.489	0.000	
We have been able to listen to each other and respect our differences of opinion	Between Groups	137.417	2	68.709	64.896	0.000	P vs. PwC	0.263	0.000	0.07
	Within Groups	1849.651	1747	1.059			P vs. R	0.687	0.000	
	Total	1987.068	1749				PwC vs. R	0.424	0.000	
We had pleasant intimate interactions	Between Groups	72.364	2	36.182	26.253	0.000	P vs. PwC	0.490	0.000	0.03
	Within Groups	2407.750	1747	1.378			PwC vs. R	−0.349	0.000	
	Total	2480.115	1749							
We shared responsibility	Between Groups	165.387	2	82.693	85.301	0.000	P vs. R	0.661	0.000	0.09
	Within Groups	1693.5858	1747	0.969			PwC vs. R	0.615	0.000	
	Total	1858.972	1749							
We have been patient enough to tolerate some annoying character from each other	Between Groups	103.454	2	51.727	58.807	0.000	P vs. PwC	0.216	0.000	0.06
	Within Groups	1536.654	1747	0.880			P vs. R	0.593	0.000	
	Total	1640.108	1749				PwC vs. R	0.377	0.000	
We agreed to make the sacrifices and renunciations that the new situation has entailed	Between Groups	74.531	2	37.265	49.036	0.000	P vs. R	0.436	0.000	0.05
	Within Groups	1327.661	1747	0.760			PwC vs. R	0.420	0.000	
	Total	1402.192	1749							

**Table 3:** Negative relationship cohabitation scale: “In the last month, with the partner I live with…”, group comparison. Note: PwC = partners with children; R = relatives; P = partner.

ANOVA										
		Sum of Squares	df	Mean Square	F	Sig.	Multiple Comparison	Mean Difference	Sig.	η^2^
We have had verbal arguments and disagreements	Between Groups	22.207	2	11.103	15.339	0.000	P vs. PwC	−0.138	0.023	0.02
	Within Groups	1264.633	1747	0.724			P vs. R	−0.282	0.000	
	Total	1286.839	1749				PwC vs. R	−0.144	0.008	
There have been barriers and obstacles between us	Between Groups	24.987	2	12.494	12.815	0.000	P vs. PwC	−0.252	0.000	0.01
	Within Groups	1703.150	1747	0.975			P vs. R	−0.280	0.000	
	Total	1728.138	1749							
Matters of power and responsibility have emerged	Between Groups	34.871	2	17.436	16.709	0.000	P vs. R	−0.342	0.000	0.02
	Within Groups	1823.001	1747	1.044			PwC vs. R	−0.224	0.000	
	Total	1857.872	1749							
Mutual trust has been compromised	Between Groups	30.048	2	15.024	16.878	0.000	P vs. PwC	−0.155	0.021	0.02
	Within Groups	1555.111	1747	0.890			P vs. R	−0.327	0.000	
	Total	1585.159	1749				PwC vs. R	−0.172	0.003	
Distance and indifference have increased	Between Groups	15.371	2	7.686	7.019	0.001	P vs. PwC	−0.230	0.001	0.01
	Within Groups	1912.821	1747	1.095			P vs. R	−0.180	0.013	
	Total	1928.192	1749							
Our differing views were unbearable	Between Groups	46.288	2	23.144	20.906	0.000	P vs. PwC	−0.181	0.014	0.02
	Within Groups	1933.992	1747	1.107			P vs. R	−0.405	0.000	
	Total	1980.279	1749				PwC vs. R	−0.224	0.000	
Intimacy interactions have been unsatisfactory	Between Groups	30.945	2	15.473	9.295	0.000	P vs. PwC	−0.189	0.049	0.01
	Within Groups	2908.166	1747	1.665			PwC vs. R	0.311	0.000	
	Total	2939.111	1749							
Certain annoying traits about the other have turned out to be intolerable	Between Groups	76.290	2	38.145	32.879	0.000	P vs. R	−0.497	0.000	0.04
	Within Groups	2026.808	1747	1.160			PwC vs. R	−0.354	0.000	
	Total	2103.098	1749							
Violent verbal contrasts have come between us	Between Groups	38.473	2	19.237	22.587	0.000	P vs. R	−0.349	0.000	0.03
	Within Groups	1487.840	1747	0.852			PwC vs. R	−0.259	0.000	
	Total	1526.314	1749							
Verbal violence almost turned into physical violence	Between Groups	6.140	2	18.018	19.605	0.000	P vs. R	−0.136	0.000	0.01
	Within Groups	486.146	1747	0.278			PwC vs. R	−0.109	0.000	
	Total	492.286	1749							

**Table 4:** Time perspective inventory, group comparison. Note: PwC = partners with children; R = relatives; P = partner.

ANOVA										
		Sum of Squares	df	Mean Square	F	Sig.	Multiple Comparison	Mean Difference	Sig.	η^2^
When I want to achieve something, I set goals and consider specific means for reaching those goals.	Between Groups	0.775	2	0.387	0.668	0.513				0.00
	Within Groups	989.811	1707	0.580						
	Total	990.585	1709							
I try to live my life as fully as possible one day at a time.	Between Groups	2.343	2	1.171	1.047	0.351				0.00
	Within Groups	1907.196	1705	1.119						
	Total	1909.539	1707							
If things do not get done on time, I do not worry about it.	Between Groups	19.246	2	9.623	8.431	0.000	PwC vs. P	−0.190	0.013	0.01
	Within Groups	1944.956	1704	1.141			PwC vs. R	0.239	0.000	
	Total	1964.202	1706							
I believe that my future is beautiful and well planned.	Between Groups	60.793	2	30.396	29.984	0.000	P vs. PwC	−0.176	0.014	0.03
	Within Groups	1727.458	1704	1.014			P vs. R	0.263	0.000	
	Total	1788.251	1706				PwC vs. R	0.439	0.000	
It does not make sense to worry about the future since there is nothing to do about it anyway.	Between Groups	3.637	2	1.819	1.769	0.171				0.00
	Within Groups	1751.182	1703	1.028						
	Total	1754.819	1705							
I feel that it’s more important to enjoy what you’re doing than to get work done on time.	Between Groups	0.144	2	0.072	0.063	0.939				0.00
	Within Groups	1933.559	1700	1.137						
	Total	1933.703	1702							
It gives me pleasure to think about my past.	Between Groups	2.432	2	1.216	1.201	0.301				0.00
	Within Groups	1721.072	1700	1.012						
	Total	1723.503	1702							
I do things impulsively and I take decisions at the moment.	Between Groups	7.627	2	3.813	4.292	0.014	P vs. PwC	−0.161	0.018	0.01
	Within Groups	1510.427	1700	0.888						
	Total	1518.054	1702							
I take risks to put excitement in my life.	Between Groups	16.834	2	8.417	10.055	0.000	P vs. R	−0.246	0.000	0.01
	Within Groups	1423.099	1700	0.837			PwC vs. R	−0.140	0.021	
	Total	1439.933	1702							

**Table 5:** Perceived stress, group comparison. Note: PwC = partners with children; R = relatives; P = partner.

ANOVA										
		Sum of Squares	df	Mean Square	F	Sig.	Multiple Comparison	Mean Difference	Sig.	η^2^
In the last month, how often have you been upset because of something that happened unexpectedly	Between Groups	0.831	2	0.416	0.477	0.621				0.00
	Within Groups	1522.404	1747	0.871						
	Total	1523.235	1749							
In the last month, how often have you felt that you were unable to control the important things in your life	Between Groups	6.118	2	3.059	3.068	0.047	PwC vs. R	−0.138	0.042	0.00
	Within Groups	1742.093	1747	0.997						
	Total	1748.211	1749							
In the last month, how often have you felt nervous and “stressed”	Between Groups	28.411	2	26.499	16.226	0.000	P vs. R	−0.289	0.000	0.02
	Within Groups	1529.452	1747	0.652			PwC vs. R	−0.239	0.000	
	Total	1557.863	1749							
In the last month, how often have you felt confident about your ability to handle your personal problems	Between Groups	44.996	2	22.498	33.849	0.000	P vs. R	0.291	0.000	0.04
	Within Groups	1161.143	1747	0.665			PwC vs. R	0.356	0.000	
	Total	1206.139	1749							
In the last month, how often have you felt that things were going your way	Between Groups	52.999	2	26.499	40.668	0.000	P vs. R	0.316	0.000	0.04
	Within Groups	1138.359	1747	0.652			PwC vs. R	0.387	0.000	
	Total	1191.358	1749							
In the last month, how often have you found that you could not cope with all the things that you had to do	Between Groups	16.111	2	8.056	9.678	0.000	P vs. R	−0.202	0.001	0.01
	Within Groups	1454.141	1747	0.832			PwC vs. R	−0.196	0.000	
	Total	1470.252	1749							
In the last month, how often have you been able to control irritations in your life	Between Groups	28.914	2	14.457	21.929	0.000	P vs. R	0.287	0.000	0.02
	Within Groups	1151.706	1747	0.659			PwC vs. R	0.247	0.000	
	Total	1180.619	1749							
In the last month, how often have you felt that you were on top of things	Between Groups	26.361	2	13.181	18.064	0.000	P vs. R	0.247	0.000	0.02
	Within Groups	1274.738	1747	0.730			PwC vs. R	0.259	0.000	
	Total	1301.099	1749							
In the last month, how often have you been angered because of things that were outside of your control	Between Groups	29.780	2	14.890	16.832	0.000	P vs. R	−0.231	0.000	0.02
	Within Groups	1545.398	1747	0.885			PwC vs. R	−0.292	0.000	
	Total	1575.178	1749							
In the last month, how often have you felt difficulties were piling up so high that you could not overcome them	Between Groups	36.036	2	18.018	19.605	0.000	P vs. R	−0.286	0.000	0.02
	Within Groups	1605.561	1747	0.919			PwC vs. R	−0.305	0.000	
	Total	1641.598	1749							

**Table 6:** Coping orientation to emotions and problems, group comparison. Note: PwC = partners with children; R = relatives; P = partner.

ANOVA										
		Sum of Squares	df	Mean Square	F	Sig.	Multiple Comparison	Mean Difference	Sig.	η^2^
I have been saying to myself “this is not real”	Between Groups	16.422	2	3.059	3.068	0.003	P vs. PwC	−0.243	0.002	0.01
	Within Groups	2383.272	1740	0.997			P vs. R	−0.173	0.042	
	Total	2399.694	1742							
I concentrate my efforts on doing something about it	Between Groups	49.483	2	24.741	42.896	0.000	P vs. PwC	−0.181	0.000	0.05
	Within Groups	1002.997	1739	0.577			P vs. R	0.214	0.000	
	Total	1052.480	1741				PwC vs. R	0.395	0.000	
I look for something good in what is happening	Between Groups	22.145	2	11.072	12.241	0.000	P vs. R	0.165	0.012	0.01
	Within Groups	1572.997	1739	0.905			PwC vs. R	0.262	0.000	
	Total	1595.142	1741							
I think about how I might best handle the problem	Between Groups	21.750	2	10.875	16.931	0.000	P vs. R	0.194	0.000	0.02
	Within Groups	1116.930	1739	0.642			PwC vs. R	0.251	0.000	
	Total	1138.680	1741							
I try to get emotional support from friends or relatives	Between Groups	36.615	2	18.307	14.712	0.000	P vs. R	−0.226	0.002	0.02
	Within Groups	2163.952	1739	1.244			PwC vs. R	−0.334	0.000	
	Total	2200.567	1741							
I think hard about what steps to take	Between Groups	3.709	2	1.855	2.046	0.130				0.00
	Within Groups	1576.186	1739	0.906						
	Total	1579.895	1741							
I pray more than usual	Between Groups	59.639	2	29.819	20.129	0.000	P vs. PwC	−0.372	0.000	0.02
	Within Groups	2573.232	1737	1.481			PwC vs. R	0.398	0.000	
	Total	2632.871	1739							
I try to get advice from someone about what to do	Between Groups	18.012	2	9.006	9.696	0.000	P vs. R	−0.184	0.005	0.01
	Within Groups	1613.319	1737	0.929			PwC vs. R	−0.226	0.000	
	Total	1631.330	1739							
